# Strongest Angle-of-Arrival Estimation for Hybrid Millimeter Wave Architecture with 1-Bit A/D Equipped at Transceivers

**DOI:** 10.3390/s22093140

**Published:** 2022-04-20

**Authors:** Ruihan Li, Lou Zhao, Chunshan Liu, Meihua Bi

**Affiliations:** School of Communication Engineering, Hangzhou Dianzi University, Hangzhou 310018, China; ryan@hdu.edu.cn (R.L.); chunshan.liu@hdu.edu.cn (C.L.); bmhua@hdu.edu.cn (M.B.)

**Keywords:** hybrid, millimeter wave, 1-bit A/Ds

## Abstract

This paper proposes an effective strongest angles of arrival (AoAs) estimation algorithm for a hybrid millimeter wave (mmWave) communication system with 1-bit analog-to-digital/digital-to-analog converters (A/Ds) equipped at transceivers. The proposed algorithm aims to reduce the required number of estimation overheads, while maintaining the root mean square error (RMSE) of strongest AoA estimates at the base station. We obtain the quantization thresholds of A/Ds for different signal-to-noise ratios (SNRs) and numbers of antennas via numerical simulations, based on which, the strongest AoAs can be estimated with a small amount of overheads. The proposed algorithm is compared with conventional schemes including 1-bit FFT and 1-bit exhaustive search, as well as 1-bit Cramér-Rao lower bound. Simulation results verify the effectiveness of our proposed algorithm in terms of reducing estimation overheads while maintaining reasonable estimation performance in low SNRs.

## 1. Introduction

Millimeter wave (mmWave) communication can offer high data rates and low latency for outdoor and indoor cellular systems and hence is an important candidate in the fifth generation (5G) mobile communications [1,2,3]. In mmWave communications, a large number of antennas with beamforming are required to overcome the high propagation path loss [4]. However, the hardware complexity, energy consumption, and channel estimation overhead generally increase with the number of antennas, which impose constraints on the application of mmWave communication [5,6].

To reduce the power consumption and hardware complexity, multiple solutions have been proposed. One promising technique is the hybrid architecture of multiple-input multiple-output (MIMO), which uses a reduced number of radio frequency (RF) chains at transceivers compared to the fully digital one that has the same number of RF chains to that of the antennas [7,8]. The analog-digital hybrid architecture can achieve a considerable array gain in the analog domain via analog beamforming and meanwhile manage multi-stream interference via baseband digital precoding. As have been investigated in the literature, effective analog beamforming and digital precoding algorithms can achieve a considerable rate performance and a higher energy efficiency compared to fully-digital architecture [8,9,10,11].

Another potential technique to reduce the power consumption and hardware complexity is utilizing low-resolution analog-to-digital/digital-to-analog converters (A/Ds) at transceivers. Since the power consumption of A/Ds increases exponentially with the number of quantization bits, utilizing low-resolution A/Ds can significantly reduce the power consumption [12,13,14]. In the literature, researches on massive MIMO systems with low-resolution A/Ds have focused on various aspects including nonlinear quantization errors [15], channel estimation errors [16,17,18], precoding error propagation [19,20], etc.

To further reduce the power consumption and hardware complexity of mmWave communication systems, one natural solution is to combine the hybrid architecture with low-resolution A/Ds [12,21]. For the hybrid architecture with low-resolution A/Ds, one of the most critical problems is to identify the dominant multipath components of the channel, i.e., estimating the strongest angles of arrival (AoAs) [22], where conventional mmWave channel estimation algorithms are not applicable.

In the literature, one popular approach to estimate the strongest AoAs of mmWave channels is to use spatial-scanning based beam search [23,24,25]. To reduce the overhead of beam search and ensure high AoA estimation accuracy, various algorithms have been proposed [26,27,28]. However, compared to scenarios that utilizing low-resolution A/Ds, these algorithms, e.g., [26,27,28], assuming high-resolution A/Ds at transceivers will lead to a significant higher energy consumption.

For mmWave channel estimation with low-resolution A/Ds, the related works are relatively limited [29,30] due to its challenging nature. The existing solutions in [29,30] mainly work in moderate to signal-to-noise ratio (SNR) scenarios, while low SNR scenarios are common especially in outdoor mmWave communications, due to the high propagation loss of mmWave signals.

To address the channel estimation problem at low SNRs with 1-bit A/Ds, we first propose a channel estimation algorithm for hybrid architecture with 1-bit A/Ds equipped at transceivers. The proposed algorithm exploits Wald-type confidence interval and detection probability to design a new reward function to reduce the estimation overheads while maintaining the root mean square error (RMSE) performance of the estimation of strongest AoA components. In addition, we obtain the semi-analytical relationship between the receive SNR, the designed practical quantization thresholds, and the average estimation overheads. Also, we compare our proposed algorithm with conventional benchmarks, e.g., the 1-bit FFT algorithm and the approximated Cramér-Rao lower bound (CRLB) for hybrid architecture equipped with 1-bit A/Ds. Numerical results show that the proposed algorithm can obtain a reasonable AoA estimation performance and reduce the estimation overhead at the same time, especially in low SNR regimes.

Notation: $E_{h}\left(\cdot \right)$ denotes statistical expectation operation with respect to random variable *h*, $C^{M\times N}$ denotes the space of all M×N matrices with complex entries; ${\left(\cdot \right)}^{-1}$ denotes inverse operation; ${\left(\cdot \right)}^{H}$ denotes Hermitian transpose; ${\left(\cdot \right)}^{*}$ denotes complex conjugate; ${\left(\cdot \right)}^{T}$ denotes transpose; |·| denotes the absolute value of a complex scalar; The distribution of a circularly symmetric complex Gaussian (CSCG) random vector with a mean vector x and a covariance matrix $\sigma^{2}I$ is denoted by $\mathrm{CN}(x,\sigma^{2}I)$, and ∼ means “distributed as”. $I_{P}$ is an P×P identity matrix.

## 2. System Model

**Figure 1:**
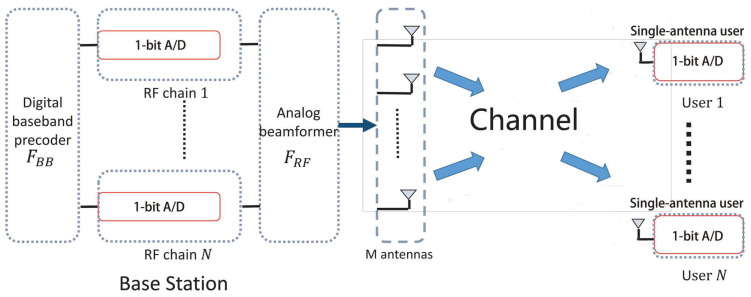
The considered hybrid mmWave system with 1-bit A/D equipped at transceivers for strongest AoA components estimation.

In this work, we consider a hybrid mmWave system in which an *M*-antenna $N_{\mathrm{RF}}$-RF-chain base station (BS) wishes to communicate with *N* single-RF-chain single-antenna users, e.g., $M⩾N_{\mathrm{RF}}⩾N⩾1$. We assume that the MU mmWave system is operating in a time division duplex (TDD) mode. In addition, the BS and users are fully synchronized [31]. The BS is equipped with a fully connected hybrid architecture and a uniform linear array (ULA), where each RF chain is connected to the *M* antennas. We also consider that 1-bit A/Ds are equipped at both transceivers, as illustrated in Figure 1.

We consider a narrowband block fading mmWave channel with one dominate path and $N_{\mathrm{cl}}$ scattering components [32,33,34,35,36]. With this model, the uplink channel $h_{k}$ can be represented as

(1) $$h_{k}=\underset{\mathrm{Strongest}\;\mathrm{component}}{\underset{︸}{\sqrt{\frac{\varsigma_{k}}{\varsigma_{k}\;+\;1}}h_{k,\mathrm{LOS}}}}+\underset{\mathrm{Scattering}\;\mathrm{components}}{\underset{︸}{\sqrt{\frac{1}{\varsigma_{k}+1}}\sqrt{\frac{1}{N_{\mathrm{cl}}}}\overset{N_{\mathrm{cl}}}{\sum_{l=1}}{\hat{\alpha }}_{k,l}h_{k,l}}},$$

where ${\hat{\alpha }}_{k,l}$, $l\in \{1,\cdots ,N_{\mathrm{cl}}\}$, represents the complex small-scale fading for the *l*-th scattering component [37], which we assume to follow a complex Gaussian distribution with zero mean and unit variance (Note that, the power ratio of the strongest path power over the sum of other scattered paths’ power, $\varsigma_{k}=1/\left[\sum_{i=1}^{N_{\mathrm{cl}}}{\left|{\tilde{\alpha }}_{k,i}\right|}^{2}\right]$, is usually larger than 1 [37]. In an outdoor scenario, the power ratio for line-of-sight (LOS) can be 10 dB and the power ratio for none-line-of-sight (NLOS) can be 6 dB [37]). In addition, $h_{k,l}\in C^{M\times 1}$ are the array response vectors of user *k* at the BS associated to the *l*-th propagation path, which can be expressed as

(2) $$h_{k,l}={\left[1,e^{-j2\pi \frac{d}{\lambda }\cos\left(\theta_{k,l}\right)},\ldots ,e^{-j2\pi \left(M-1\right)\frac{d}{\lambda }\cos\left(\theta_{k,l}\right)}\right]}^{T},$$

where *d* is the antenna spacing at the BS and λ is the carrier wavelength. Variable $\theta_{k,l}$ is the AoA of the *l*-th path at the antenna array of the BS from user *k*, which is assumed to be uniformly distributed between $\left[0,\;\pi \right]$.

To estimate the AoAs at the BS, each user transmits pilots respectively according to a fixed “0–1” codebook while the BS performs periodic beam scan to measure pilot signals [27]. The goal of this process is to find the AoAs of the dominant paths such that beams can be steered to these directions to achieve the best effective SNR. Denoting $F=\{f_{\mathrm{RF},k,1},\ldots ,f_{\mathrm{RF},k,F}\}$ as the BS codebook [28], the best beamforming vector for the strongest AoA estimation can be expressed as

(3) $$\begin{array}{c} f_{\mathrm{RF},k}^{H}=\arg\max_{f_{\mathrm{RF},k,i}\in F}\left|f_{\mathrm{RF},k,i}^{H}h_{k}\right|, \end{array}$$

where $f_{\mathrm{RF},k}^{H}$ is the best beam of the BS, and $f_{\mathrm{RF},k,i}$ is the beam vector for spatial scanning of the *i*-th direction. With ULA, the beamformer, $f_{\mathrm{RF},k,i}$, k∈{1,…,N}, can be represented as

(4) $$\begin{array}{c} f_{\mathrm{RF},k,i}=\frac{1}{M}{\left[\;\begin{array}{c} 1,e^{-j2\pi \frac{d}{\lambda }\mu },\ldots ,e^{-j2\pi \left(M-1\right)\frac{d}{\lambda }\mu } \end{array}\;\right]}^{T}\;, \end{array}$$

where $\mu =\cos\left({\bar{\theta }}_{i}\right)=-1+\frac{2i-1}{M}\in \left(-1,1\right),i\in \left\{1,\ldots ,M\right\}$.

Denote further $h_{k,i}=f_{\mathrm{RF},k,i}^{H}h_{k}$ as the effective channel after receiving beamforming $f_{\mathrm{RF},k,i}^{H}$. Then the signal before quantization received at *i*-th RF chain of the BS from user *k* can be expressed as

(5) $$y_{k,i}=h_{k,i}s_{k}+f_{\mathrm{RF},k,i}^{H}z,$$

where $s_{k}$ is the finite-alphabet signal with covariance $E\left[s_{k}s_{k}^{H}\right]=E_{s}=1$, k∈{1,…,N}, $E_{s}$ is the average symbol energy transmitted from user *k*, $h_{k}\in C^{M\times 1}$, k∈{1,…,N}, is the channel vector between the BS and user *k*, and $z\in C^{M\times 1}∼\mathrm{CN}\left(0,\sigma^{2}I\right)$ is the additive noise assuming to be complex white Gaussian with zero-mean variance $\sigma^{2}$.

The received signal after 1-bit quantization can be represented as:

(6) $$\begin{array}{cc} |{\hat{y}}_{k,i}| & =Q_{\mathrm{BS}}\left[|y_{k,i}|\right]=Q_{\mathrm{BS}}\left[|h_{k,i}Q_{\mathrm{users}}\left(s_{k}\right)+f_{\mathrm{RF},k,i}^{H}z|\right]\\ \; & =\mathrm{sign}\left[|y_{k,i}|-\tau \right]=\left\{\begin{array}{c} 1\;\;\;\;\;\;\;(|y_{k,i}|-\tau )⩾0,\\ 0\;\;\;\;\;\;\;(|y_{k,i}|-\tau )<0, \end{array}\right. \end{array}$$

where $Q_{\mathrm{BS}}\left(\cdot \right)$ and $Q_{\mathrm{users}}\left(\cdot \right)$ are 1-bit quantization operations at the BS and users, respectively. Parameter τ is the quantization threshold. Conditioned on any underlying beamforming $f_{\mathrm{RF},k,i}$ and the quantization threshold, ${\hat{y}}_{k,i}$ follows a Bernoulli distribution, for which the probability mass function (PMF) over possible outcomes *r* can be expressed as

(7) $$f_{\mathrm{pmf}}\left(i,r,p\right)=\left\{\begin{array}{cc} p_{i} & r=1,\\ q_{i}=1-p_{i} & r=0, \end{array}\right.$$

where $p_{i}\in \left[0,1\right]$ denotes the yield value that need to be estimated.

## 3. Proposed Strongest AoA Components Estimation Algorithm

### 3.1. Problem Formulation

According to (6), $p_{i}=\mathrm{Pr}\{|y_{k,i}\left|>\tau \right\}$. Clearly, the stronger the effective channel after beamforming, i.e., $|h_{k,i}|$, the higher value is $p_{i}$. Hence, the best beam identification problem in (3) becomes to identify the beam with the highest value of $p_{i}$.

Suppose *L* observations have been made by the BS at each RF chain: ${\left\{{\hat{y}}_{k,i}\right\}}_{n=1}^{L}$. Denote $I_{+}=\left\{K|{\left({\hat{y}}_{k,i}\right)}_{j}=1,j=1,\cdots ,K\right\}$ and $I_{-}=\left\{L-K|{\left({\hat{y}}_{k,i}\right)}_{t}=0,t=1,\cdots ,L-K\right\}$, respectively, then ${\hat{p}}_{i}$ can be estimated as

(8) $${\hat{p}}_{i}=\frac{K}{L},$$

where ${\hat{p}}_{i}$ can be obtained as close as $p_{i}$ provided that the number of observations *L* is sufficiently large. Therefore, in principle, the best beam can be identified by choosing the beam that has the largest ${\hat{p}}_{i}$, i∈{1,…,M}, regardless of the quantization threshold τ. However, the value of τ will affects the discrimination between the best beam and others, i.e., affecting the differences of $p_{i}$, thus will have a significant impact on the number of observations needed to identify the best beam.

Unfortunately, it is difficult to directly obtain the optimal threshold $\tau_{\mathrm{opt}}$ as well as the minimum estimation overhead without the precise knowledge of the noise variance, which is known to be hard to obtain with 1-bit A/Ds [38]. As an alternative way, one can determine the threshold τ by counting the minimum number of pilots *L* under different values of τ.

Therefore, in the remaining parts of this section, we focus on the problem of minimizing the overall AoA estimation overhead while maintaining reasonable estimation accuracy. We begin by considering how to design proper quantization thresholds to reduce the overhead in low SNR scenarios.

### 3.2. The Design of Quantization Thresholds

In this subsection, we detail the algorithm proposed for the strongest AoAs estimation with 1-bit A/Ds equipped at both transceivers. We also use the Monte Carlo simulations to statistically find appropriate quantization thresholds for different SNRs to reduce the estimation overhead. Then, the channel can be estimated with a relatively smaller number of beam searching repetitions for the case of limited hardware in the hybrid architecture with 1-bit A/Ds.

For our proposed AoAs estimation algorithm, we firstly obtain the ordered detection probability output vector through periodic beam scanning at the BS after sorting in descending order as $\left[{\hat{p}}_{(1),n},\cdots ,{\hat{p}}_{(M+1),n}\right]$, where ${\hat{p}}_{(i),n}⩾{\hat{p}}_{(i+1),n}$, ∀i. Then we calculate the Wald-type confidence interval which will be used to determine when the beam scanning stops. The confidence interval can be represented as

(9) $$\begin{array}{c} \left\{\begin{array}{c} {\hat{p}}_{(i),n}\;-\;\zeta_{(i),n}\;⩽\;{\hat{p}}_{(i),n}\;⩽\;{\hat{p}}_{(i),n}\;+\;\zeta_{(i),n},\forall i,\\ \zeta_{(i),n}=\alpha \sqrt{\frac{{\hat{p}}_{(i),n}\left(1-{\hat{p}}_{(i),n}\right)}{L}}, \end{array}\right. \end{array}$$

where α>0 is from the standard normal distribution table. In the iterative search process, a reward function λ is introduced in low SNRs to decide when to stop searching, which can be expressed as

(10) $$\begin{array}{c} \left\{\begin{array}{cc} {\hat{p}}_{(i),n}\;>{\hat{p}}_{(i+1),n},\; & \lambda =\lambda +1,\\ {\hat{p}}_{(i),n}\;-\;\zeta_{(i),n}⩾{\hat{p}}_{(i+1),n}+\;\zeta_{(i+1),n},\; & \lambda =\lambda +1. \end{array}\right. \end{array}$$

After the two aforementioned conditional judgments, the number of detection repetitions increases, L=L+1. Then, the proposed algorithm recalculate the corresponding $\zeta_{(i),n}$ and update the parameter λ. The beam search can be stopped when λ reaches to a pre-determined reward function threshold *T* (T>0). The beam with the largest ${\hat{p}}_{(i),n}$ upon termination will be selected as the best beam.

This proposed algorithm is summarized in Algorithm 1. From Algorithm 1, we calculate the number of detection repetitions required for the estimation of strongest AoA components with a given quantization threshold τ and SNR. In general, it is possible to obtain SNR via different methods [26].

From Equation (6), the quantization threshold, τ, is the only parameter during the 1-bit quantization. Though a higher quantization threshold may reduce the impact of environmental noise in low SNR regimes, it may lead to loss of useful-information since larger fractions of the received signals are quantized to 0. On the contrary, a lower quantization threshold may lead to an increase of false alarm probability (FAP) and a decrease in estimation accuracy.

In order to obtain appropriate quantization thresholds to minimize the number of detection repetitions, $L_{i}$, for different SNRs, we further introduce the SNR as a parameter in simulation to characterize the connection between τ and $L_{i}$. Our main idea is to perform statistical experiments through Monte Carlo simulation. The process for selecting specific quantization thresholds based on Monte Carlo simulation is demonstrated in Algorithm 2. With Algorithms 1 and 2, we can exhaustively search for an appropriate quantization threshold, τ, to minimize the required detection repetitions, $L_{\min}$, for a certain SNR via a large mount of digital simulations, which satisfies

(11) $$\begin{array}{c} L_{\min}⩽\left[L_{1},\cdots ,L_{i},\cdots ,L_{N}\right], \end{array}$$

where $L_{i}$ is the number of detection repetitions when the quantization threshold is set as $\tau_{i}$, $i\in \left\{1,\ldots ,N\right\}$.

To the end, with aforementioned parameters and the minimum number of detection repetitions, we can obtain the strongest AoA estimation results, ${\hat{\theta }}_{k,l}$.
**Algorithm 1** Beam Searching for Strongest AoA Components Estimation**Require:** Pre-designed analog beamforming vectors for the BS, $f_{\mathrm{RF},k}$, k∈{1,…,N}  1:Initialize n=1, $k_{i,n}=0$, the number of detection repetition L=1, and trigger condition ϖ=1  2:**while ϖ==1 do**  3:Search for M+1 beam space  4:  **for i=1:M+1**
  5:Receiving signal with $f_{\mathrm{RF},i}$, for the *i*-th direction:  6:  $y_{k,i}=f_{\mathrm{RF},k,i}^{H}h_{k}\;Q_{\mathrm{users}}\left(s_{k}\right)\;+\;f_{\mathrm{RF},k,i}^{H}z$  7:Received signal after 1-bit quantization:  8:  $|{\hat{y}}_{k,i}|=\mathrm{sign}\left[|y_{k,i}|-\tau \right]$  9:    **if $|y_{k,i}|-\tau ⩾0$**10:      $k_{i,n}=k_{i,n}+1$11:    **end**12:Calculation detection probability:13:  ${\hat{p}}_{i,n}=k_{i,n}/L$14:  **end**15:Sort the detection probability outputs $\left[{\hat{p}}_{1,n},\cdots ,{\hat{p}}_{M+1,n}\right]$ in descending order and obtain the ordered detection probability output vector as $\left[{\hat{p}}_{(1),n},\cdots ,{\hat{p}}_{(M+1),n}\right]$, where ${\hat{p}}_{(i),n}⩾{\hat{p}}_{(i+1),n}$, ∀i16:Calculate the Wald-type confidence interval by Equation (9)17:Reward function:18:Case1: Low SNR regimes19:  **if ${\hat{p}}_{(i),n}\;>{\hat{p}}_{(i+1),n}$**20:    λ=λ+121:    **if ${\hat{p}}_{(i),n}\;-\;\zeta_{(i),n}⩾{\hat{p}}_{(i+1),n}+\;\zeta_{(i+1),n}$**22:      λ=λ+123:    **end**24:  **end**25:Case2: Medium-to-high SNR regimes26:  **if ${\hat{p}}_{(i),n}\;>{\hat{p}}_{(i+1),n}$**27:    λ=λ+128:  **end**29:Stop condition:30:  **if λ>T**31:    ϖ=032:  **end**33:Repetition calculation:34:L=L+135:**end while**

### 3.3. RMSE Performance Metric

To verify the estimation accuracy of our proposed algorithm, we use the RMSE of the strongest AoA estimate as a performance metric. Meanwhile, the best achievable RMSE estimation performance benchmark with $f_{\mathrm{RF},k}$, k∈{1,…,N}, can be characterized by deriving the approximated 1-bit CRLB with additive quantization noise model (AQNM) of the antenna array [39].

The RMSE expression the AoA estimates can be represented as

(12) $$\begin{array}{c} \mathrm{RMSE}=\sqrt{\frac{1}{n}\sum_{i=1}^{n}{\left({\hat{\theta }}_{k,i}-\theta_{k}\right)}^{2}}, \end{array}$$

where $\theta_{k}$ represents the desired incidence strongest AoA component for the *k*-th user at the BS, and ${\hat{\theta }}_{k,i}$ denotes the estimated result. The RMSE of the strongest AoA component estimation proposed in our algorithm is compared to that of conventional schemes in the following simulation section.
**Algorithm 2** Monte Carlo Simulation for $L_{\min}$**Require:** Quantization threshold τ corresponding to $L_{\min}$ for different SNRs  1:Initialize τ=1, $\tau_{m}=0.5$, $L_{\min}=300$, the number of Monte Carlo simulations N=50,000  2:  low SNR regimes  3:A reasonable range for the quantization threshold  4:    **for $\tau_{n}=0.3:0.01:1$**  5:Experiment with different noise distributions  6:      **for i=1:N**  7:Run Algorithm 1 to obtain $L_{i}$ required for strongest AoA estimation for a certain $\tau_{i}$ in case 1  8:      **end**  9:Calculate the mean of $L_{i}$ with different noise distributions10:      $L_{\mathrm{ave}}=\frac{1}{N}\sum_{i=1}^{N}L_{i}$11:      **if $L_{\mathrm{ave}}⩽L_{\min}$**12:        $\tau =\tau_{n}$13:        $L_{\min}=L_{\mathrm{ave}}$14:      **end**15:    **end**16:  **end**17:  Medium-to-high SNR regimes18:    **for i=1:N**19:Run Algorithm 1 directly to obtain $L_{i}$ required for strongest AoA estimation for $\tau_{m}$ in case 220:    **end**21:    $L_{\min}=L_{\mathrm{ave}}=\frac{1}{N}\sum_{i=1}^{N}L_{i}$22:  **end**

### 3.4. Approximated 1-Bit CRLB with AQNM

In this subsection, we detail the corresponding approximated CRLB of hybrid architecture with 1-bit A/Ds, which can be used as a reference benchmark [30,40,41].

For hybrid mmWave communication system equipped with ideal A/Ds, the received signal follows a circularly complex Gaussian distribution, i.e., ${\hat{y}}_{i}∼\mathrm{CN}\left(a\left(\theta \right)s_{k},\sigma^{2}I\right)$. In addition, we can obtain the array response for the *m*-th element of the ULA as follows

(13) $$\begin{array}{c} {\dot{a}}_{m}\left(\theta \right)=e^{j2\pi \frac{d}{\lambda }m\cos\left(\theta \right)}i2\pi \frac{d}{\lambda }m\sin\left(\theta \right), \end{array}$$

and

(14) $$\begin{array}{cc} {\left|\left|\dot{a}\left(\theta \right)\right|\right|}^{2} & =\sum_{m=0}^{M-1}{\left|{\dot{a}}_{m}\left(\theta \right)\right|}^{2}={\left(2\pi \frac{d}{\lambda }\sin\left(\theta \right)\right)}^{2}\sum_{m=1}^{M-1}m^{2}\\ \; & ={\left(2\pi \frac{d}{\lambda }\sin\left(\theta \right)\right)}^{2}\frac{M(M-1)(2M-1)}{6}. \end{array}$$

Hence, we can further calculate the Fisher Information Matrix (FIM) as

(15) $$\begin{array}{cc} \mathrm{FIM} & =\frac{2}{\sigma^{2}}ℜ\left\{\;\frac{\partial a\left(\theta \right)s_{k}}{\partial \theta }{\left(\;\frac{\partial a\left(\theta \right)s_{k}}{\partial \theta }\;\right)}^{H}\;\right\}=2\frac{P}{\sigma^{2}}{\left|\left|\dot{a}\left(\theta \right)\right|\right|}^{2}\\ \; & =2\mathrm{SNR}{\left(2\pi \frac{d}{\lambda }\sin\left(\theta \right)\right)}^{2}\frac{M(M\;-\;1)(2M\;-1)}{6}, \end{array}$$

where $\mathrm{SNR}=\frac{P}{\sigma^{2}}$, and P=1.

Based on the obtained FIM, the corresponding $\mathrm{CRLB}=\frac{1}{L\cdot \mathrm{FIM}}$ expression for ideal quantization can be expressed as

(16) $$\begin{array}{c} \mathrm{CRLB}=\frac{3\lambda^{2}}{4\pi^{2}d^{2}L\sin^{2}\left(\theta \right)M\left(M\;-\;1\right)\left(2M\;-\;1\right)\mathrm{SNR}}. \end{array}$$

Utilizing AQNM, we can further derive the approximation of CRLB for hybrid architecture equipped with 1-bit A/Ds at transceivers [41], which is given by

(17) $$\begin{array}{cc} {\mathrm{CRLB}}_{1b}= & \frac{1+(1-\eta )\mathrm{SNR}}{\eta }\mathrm{CRLB}\\ = & \frac{3\lambda^{2}+1.0902\lambda^{2}\mathrm{SNR}}{2.5464\pi^{2}d^{2}L\sin^{2}\left(\theta \right)M\left(M-1\right)\left(2M-1\right)\mathrm{SNR}}, \end{array}$$

where 1−η is the inverse of signal-to-quantization-noise ratio (SQNR) (approximately η=0.6366 for 1-bit quantization [42]). Hence, the obtained approximated 1-bit CRLB of hybrid architecture can be used as a RMSE performance benchmark in simulations.

## 4. Simulation Results and Discussions

In this section, we verify the effectiveness of our proposed algorithms via simulation results.

First, we demonstrate the relationship between the quantization threshold, τ, and the minimum required number of detection repetitions, $L_{\min}$, for different SNRs. In addition, we also illustrate the RMSE performance versus SNR with different detection repetitions for different scenarios. Without further clarification, the detailed parameters in simulations are set as follows: K-factor =10, the number of RF-chains equipped at the BS is $N_{\mathrm{RF}}=N$, the number of users, N=5, the number of ULA antennas, M=64, the reward function threshold, T=3, and the receive SNR before beamforming ranges from −25 dB to −5 dB.

**Figure 2:**
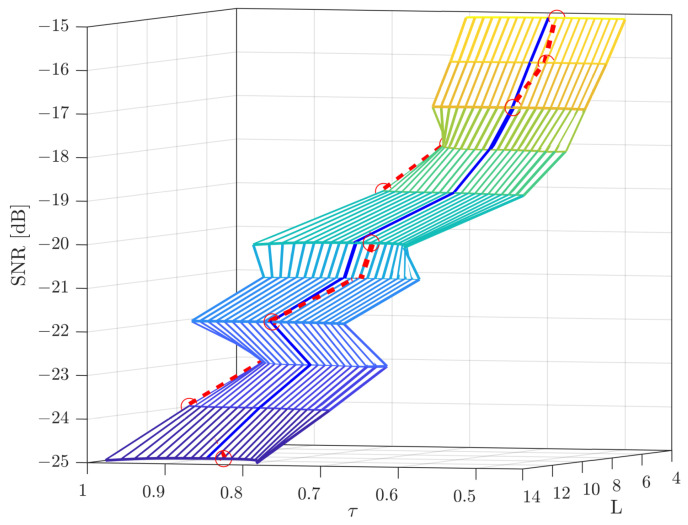
A demonstration for quantization threshold, τ, and detection repetitions, *L*, versus SNR [dB] with M=64.

Figure 2 illustrates the number of detection repetitions $L_{\min}$ required for our proposed beamforming direction search algorithm versus different quantization thresholds τ for M=64 in low SNR regimes. We also set α=1.96 in Equation (9) to calculate the 95% confidence intervals as a trade-off between estimation accuracy and overhead. According to characteristics of mmWave communication systems, we choose SNR =−15 dB as the conversion point, i.e., SNR ⩽−15 dB will be considered as low SNR regimes.

In Figure 2, it can be seen that the the required $L_{i}$ changes with different $\tau_{i}$ for the strongest AoA components estimation. Thus, it is interesting to choose a proper τ to minimize the number of required detection repetitions $L_{\min}$ via comprehensive Monte Carlo simulations, which are illustrated in Table 1 for different SNRs with M=64 antennas. Meanwhile, according to the results presented in Table 1, we can note that both τ and $L_{\min}$ decrease with an increasing SNR.

**Figure 3:**
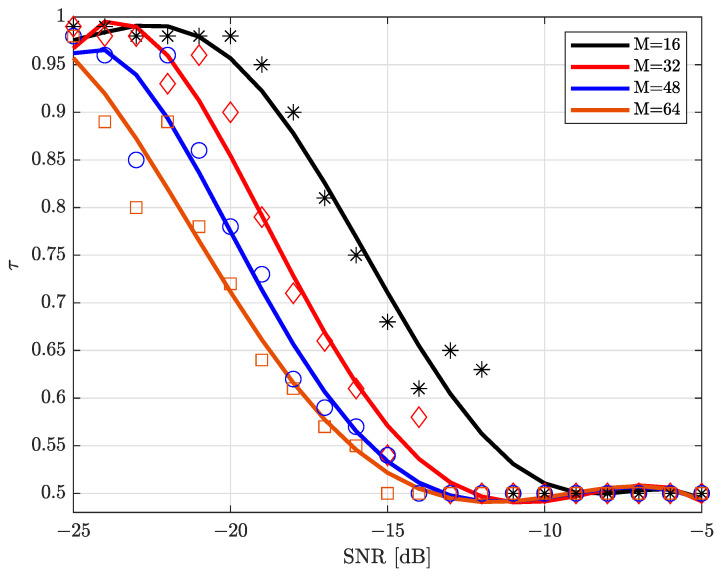
Quantization threshold, τ, versus SNR [dB] for different number of antennas.

Figure 3 illustrates the variation of τ versus SNR for different numbers of antennas, e.g., M∈{16,32,48,64}. We can observe that, τ decreases from 0.98 to 0.50 in low SNR regimes. However, it stabilizes around 0.50 for medium-to-high SNR regimes, e.g., τ≈0.50. Furthermore, for a given receive SNR, the quantization threshold, τ, decreases with an increasing number of antennas M as extra beamforming gain can be provided.

Based on simulation results given in Figure 3, we can further approximate the relationship between τ, SNR, and *M* as follows:

(18) $$\begin{array}{c} \tau \approx \left\{\begin{array}{cc} 0.98, & \mathrm{SNR}<-19-\frac{M}{8},\\ \left(-0.033-\frac{24}{125M}\right)\cdot \mathrm{SNR}+\frac{48}{25M},\;\;\;\;\;\;-19-\frac{M}{8}⩽ & \mathrm{SNR}⩽-7-\frac{M}{8},\\ 0.50, & \mathrm{SNR}>-7-\frac{M}{8}. \end{array}\right. \end{array}$$

We note here, results given in Equation (18) will be exploited in the following simulations.

**Figure 4:**
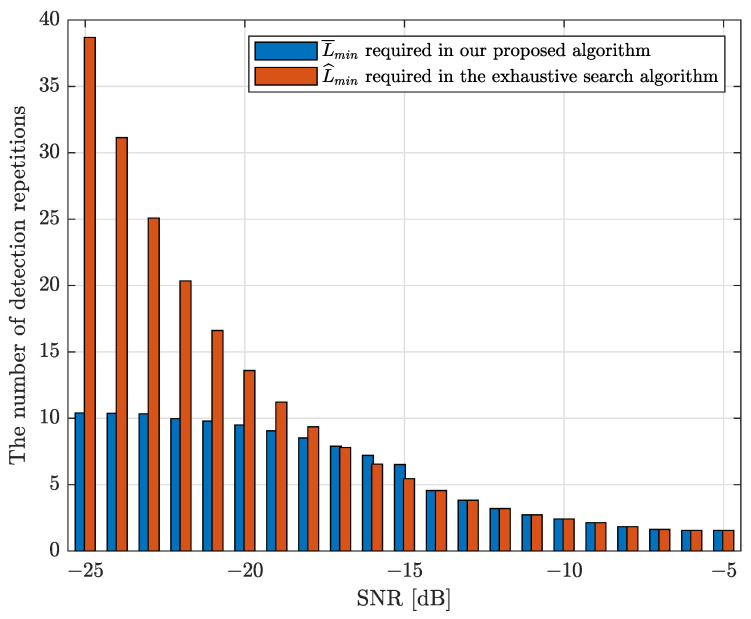
Comparison of the minimum number of detection repetitions between our proposed method and the exhaustive search algorithm, M=64.

Figure 4 compares the minimum number of detection repetitions, $L_{\min}$, obtained by utilizing the proposed algorithm with that of the conventional exhaustive search algorithm for M=64. It is clear that our proposed algorithm can significantly reduce the required number of detection repetitions in the low SNR regime, which verifies the effectiveness of our proposed algorithm.

**Figure 5:**
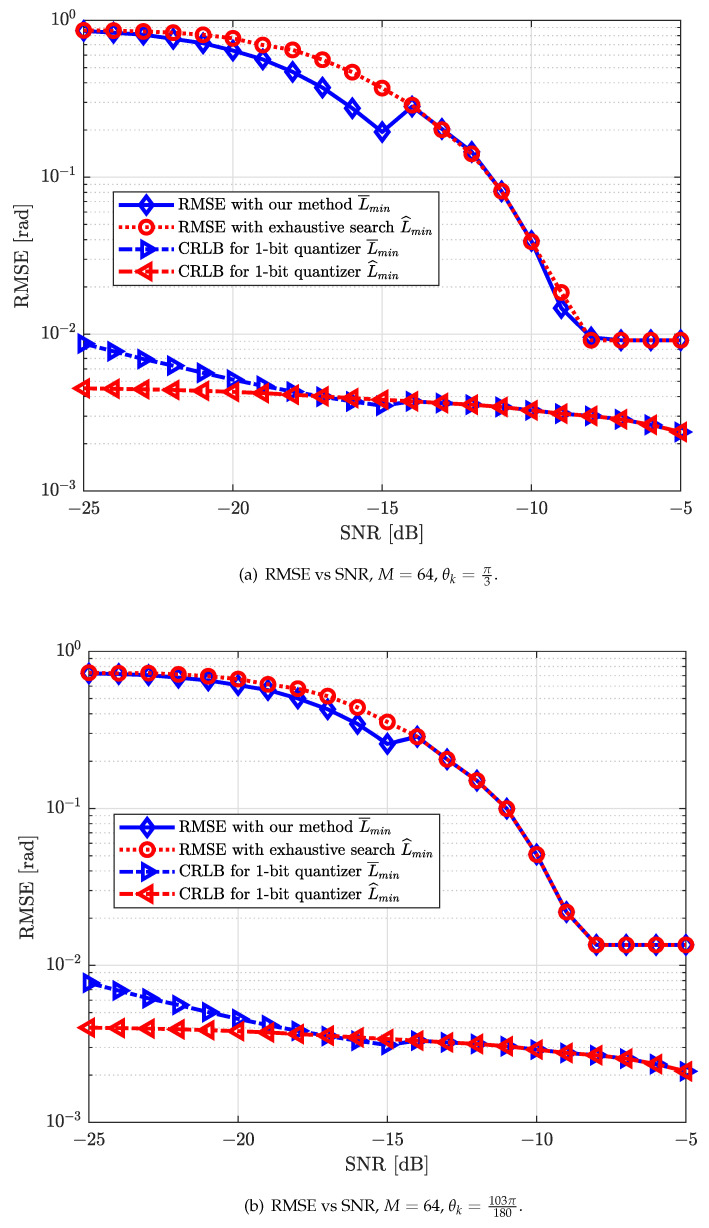
RMSE [rad] performance versus different SNRs [dB] for different scenarios.

In Figure 5, the strongest AoA estimation performance of our proposed algorithm is compared with that of the exhaustive search algorithm using RMSE as the performance metric for different scenarios in the scattering environment, e.g., $\theta_{1}=\frac{\pi }{3}$ and $\theta_{2}=\frac{103\pi }{180}$. It is interesting to see that our proposed algorithm works well for multi-path scenarios. The numbers of detection repetitions required for these two algorithms are presented in Figure 4. It can be observed that our proposed algorithm can have a slightly higher performance in terms of strongest AoA estimation while consuming a much smaller number of detection repetitions in low SNR regimes. In addition, the strongest AoA estimation performance of these two algorithms converge in medium-to-high SNR regimes. As expected, the performance of these two different algorithms approach to 1-bit CRLB with an increasing SNR, which is appropriated by using AQNM.

**Figure 6:**
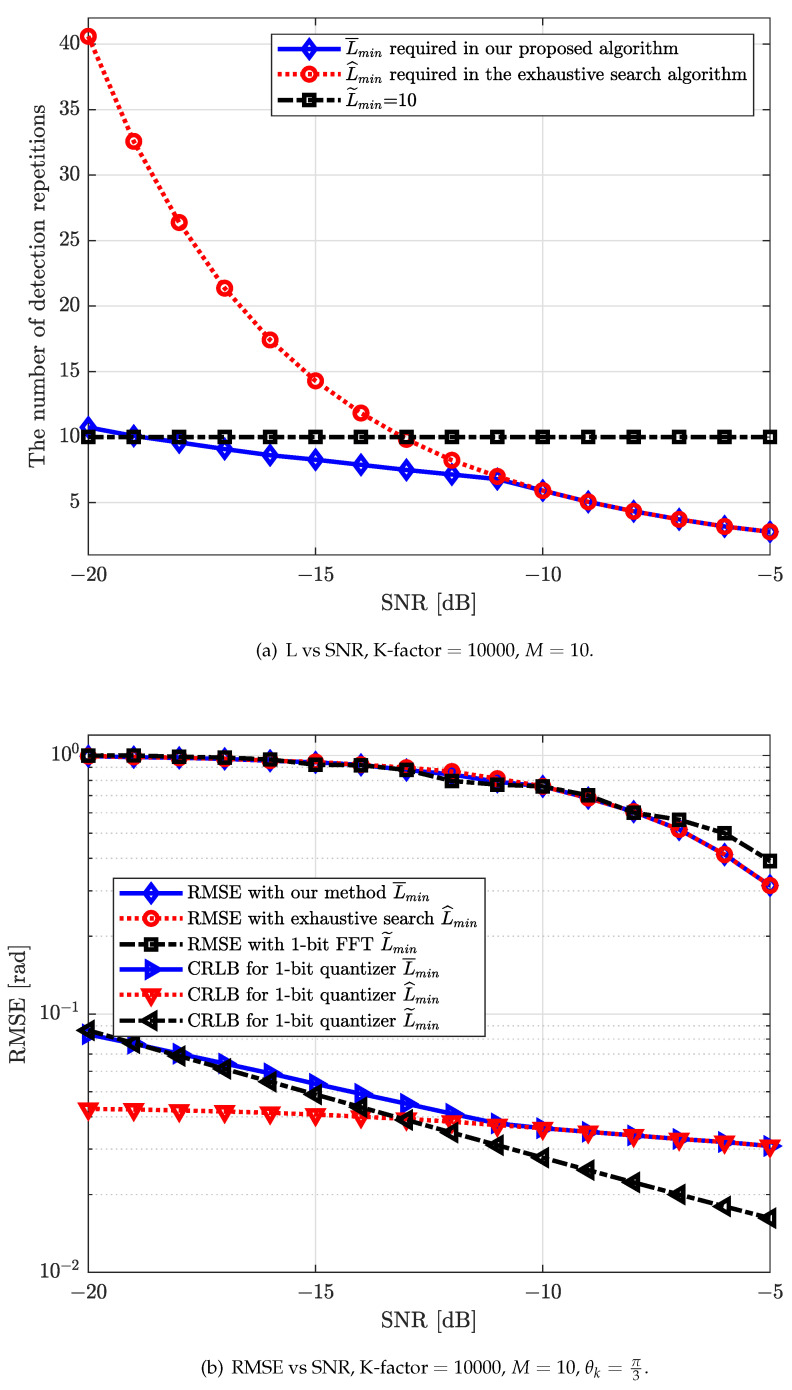
(**a**) The number of detection repetitions required for different algorithms versus different SNR for M=10; (**b**) RMSE [rad] versus different SNRs for M=10 with our proposed beamforming direction search algorithm, the exhaustive search algorithm and 1-bit FFT.

**Figure 7:**
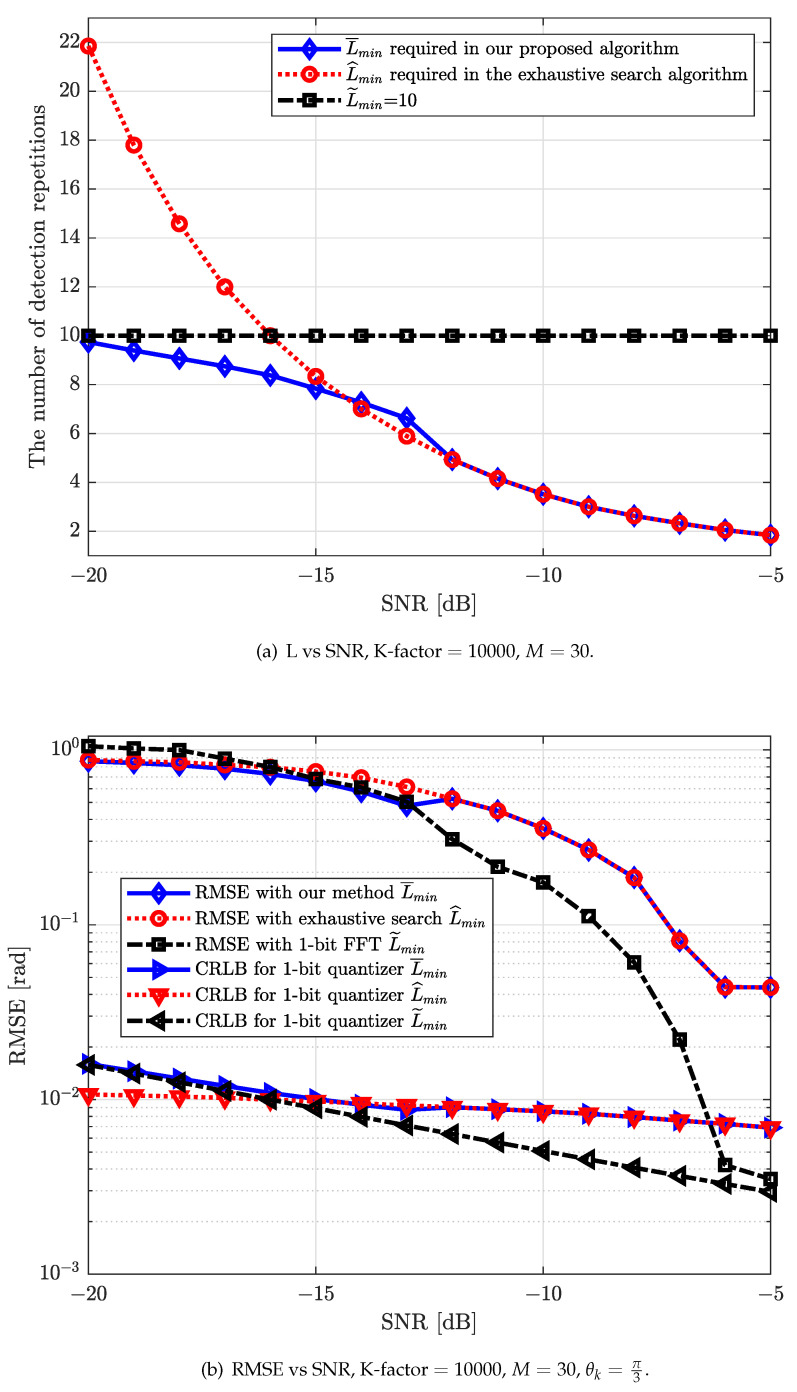
(**a**) The number of direction repetitions required for different algorithms versus different SNRs for M=30; (**b**) RMSE [rad] versus different SNRs for M=30 with our proposed beamforming direction search algorithm, the exhaustive search algorithm and 1-bit FFT.

Meanwhile, in Figure 6 and Figure 7, we calculate the performance of strongest AoA estimation with different conventional schemes for the single-path scenario for different numbers of antennas *M*, e.g., 1-bit CRLB appropriated by using AQNM and 1-bit FFT.

Figure 6b and Figure 7b compare the strongest AoA estimation performance of our proposed algorithm with that of the exhaustive search algorithm and research results from work [30] (Yoffe I et al.). The numbers of detection repetitions, $L_{\min}$, required for the strongest AoA estimation of these three algorithms are presented in Figure 6b and Figure 7a (In work [30], ${\tilde{L}}_{\min}$ required by utilizing 1-bit FFT is fixed to 10.). It can be observed in Figure 6b and Figure 7b that our proposed algorithm can effectively reduce the estimation overhead while maintaining a reasonable estimation performance when the number of antennas is small. It can also be observed that our proposed algorithm can have a slightly higher performance in terms of strongest AoA estimation compared to 1-bit FFT algorithm in low SNRs. In the medium-to-high SNR regime, although our proposed algorithm is inferior with the 1-bit FFT in terms of RMSE performance, the detection repetitions required of our proposed algorithm is significantly smaller.

## 5. Conclusions

In this paper, we proposed a novel strongest AoA estimation algorithm for hybrid mmWave communication equipped with 1-bit A/Ds at transceivers. The proposed algorithm aims to estimate the strongest AoA components while reducing the number of detection repetitions as much as possible. We conducted Monte Carlo simulation experiments to obtain appropriate quantization thresholds for different SNRs and different number of antennas M. With appropriately designed quantization thresholds, we further investigated the RMSE performance of the strongest AoA estimation of our proposed algorithm and compared to different schemes via simulations, e.g., 1-bit FFT, 1-bit CRLB, and the exhaustive search algorithm. Comprehensive simulation results verified that our proposed algorithm can significantly reduce the estimation overheads while maintaining the estimation performance of strongest AoA components in low SNR regimes.

## Figures and Tables

**Table 1:** The results of the Monte Carlo simulation for different SNRs.

Case 1: Low SNR Regimes											
SNR [dB]	−25	−24	−23	−22	−21	−20	−19	−18	−17	−16	−15
τ	0.98	0.89	0.80	0.89	0.78	0.72	0.64	0.61	0.57	0.55	0.54
$L_{\min}$	10.40	10.36	10.34	9.97	9.78	9.49	9.05	8.52	7.89	7.21	6.51
**Case 2: Medium-to-High SNR Regimes**											
SNR [dB]	−14	−13	−12	−11	−10	−9	−8	−7	−6	−5	
τ	0.50	0.50	0.50	0.50	0.50	0.50	0.50	0.50	0.50	0.50	
$L_{\min}$	4.55	3.82	3.20	2.73	2.41	2.13	1.83	1.63	1.55	1.55	

## Data Availability

Not applicable.
